# Epstein–Barr Virus (EBV) Epithelial Associated Malignancies: Exploring Pathologies and Current Treatments

**DOI:** 10.3390/ijms232214389

**Published:** 2022-11-19

**Authors:** Oren Shechter, Daniel G. Sausen, Elisa S. Gallo, Harel Dahari, Ronen Borenstein

**Affiliations:** 1 Department of Microbiology and Molecular Cell Biology, Eastern Virginia Medical School, Norfolk, VA 23501, USA; 2 Tel-Aviv Sourasky Medical Center, Division of Dermatology, Tel-Aviv 6423906, Israel; 3 The Program for Experimental and Theoretical Modeling, Division of Hepatology, Department of Medicine, Stritch School of Medicine, Loyola University Chicago, Maywood, IL 60153, USA

**Keywords:** EBV, epithelial cancer, nasopharyngeal cancer, gastric cancer, latency

## Abstract

Epstein–Barr virus (EBV) is one of eight known herpesviruses with the potential to infect humans. Globally, it is estimated that between 90–95% of the population has been infected with EBV. EBV is an oncogenic virus that has been strongly linked to various epithelial malignancies such as nasopharyngeal and gastric cancer. Recent evidence suggests a link between EBV and breast cancer. Additionally, there are other, rarer cancers with weaker evidence linking them to EBV. In this review, we discuss the currently known epithelial malignancies associated with EBV. Additionally, we discuss and establish which treatments and therapies are most recommended for each cancer associated with EBV.

## 1. Introduction

Epstein–Barr Virus (EBV) is a highly prevalent gamma herpesvirus that has infected more than 90% of the population worldwide [1]. In addition to being the causative agent of infectious mononucleosis, EBV was the first human oncogenic virus to be discovered and has been linked to numerous malignancies, including various epithelial and mesenchymal cancers and lymphomas [2]. EBV-associated cancers are known to affect both immune-competent hosts and immunocompromised patients [3]. Globally, it is believed that EBV is responsible for approximately 1.5% of all human cancers [4]. EBV transmission primarily occurs through saliva, with increased levels of viral DNA being found in salivary secretions after the initial infection [5]. Other methods of transmission include blood transfusion and allograft transplantation [6]. Socioeconomics influence the age at which primary EBV infection occurs, as demonstrated by the cohort study performed by Gares et al. in the UK. This study found that children who slept in overcrowded homes (OR = 1.14 (1.10–1.31)) were found to have a higher rate of infection with EBV by three years of age when compared to children who lived in better conditions [7].

EBV is a member of the *Herpesviridae* family; more specifically, the *Gammaherpesvirinae* subfamily. It is also known as Human herpesvirus 4 (HHV4). Its genome is composed of linear double-stranded DNA that is approximately 170 kb in length and includes approximately 85 genes [8]. Traditionally, EBV strains have been classified into type 1 and type 2 (also known as types A and B, respectively) primarily based on the sequence of their EBV nuclear antigen (EBNA), specifically EBNA2 and EBNA3A/B/C latency genes [9]. Type 1 EBV strains are more prevalent worldwide, with type 2 being more prevalent in Alaska, Papua New Guinea and Central Africa [10]. The main phenotypic difference in vitro between these two strains is that type 1 EBV transforms human B lymphocytes into lymphoblastoid cell lines (LCL) more efficiently than type 2 [9]. In a retrospective study conducted by Monteiro et al., EBV2 was shown to have a longer clinical course than EBV1, with an average duration of 17.6 days of fever (range of 1–90 days), while EBV1 had an average range of 14.8 days (range of 1–30 days) [11]. Interestingly, this study also noted that the levels of hepatic enzymes were significantly higher, on average, in EBV1-infected patients aged 14 years and older when compared to those infected with EBV2 or coinfected with EBV1 and EBV2 [11].

B lymphocytes are the primary targets of EBV infection due to their expression of complement receptor type 2 (CR2), also known as the complement C3d receptor or CD21 [12]. EBV first infects B cells through the binding of the viral envelope protein gp350 with CR2 [13]. The ensuing interaction of viral envelope proteins gp42, gH/gL and gB with the human leukocyte antigen (HLA) class II protein on the B cell surface results in the fusion of the viral envelope with the host cell membrane and allows for EBV to enter the cell [14]. Another target of EBV is epithelial cells. While EBV enters B cells by fusion with an endocytic membrane after endocytosis, EBV enters epithelial cells by fusion at the plasma membrane [15]. The glycoproteins used by EBV to enter epithelial cells depend on both the cell type and the expression of CR2. EBV uses gp350 for attachment to CR2-positive epithelial cells [16]. For CR2-negative epithelial cells, EBV can use the multi-spanning transmembrane envelope protein BMRF-2 to bind to integrin αvβ1, or it can use gH/gL to bind to integrin αvβ5, αvβ6 or αvβ8 [17,18,19].

After the initial infection, EBV establishes and maintains an episome in the nucleus of the host cell. It predominantly establishes latency that cannot be eradicated in B cells [20]. In a recent study performed by Wang et al., EBV episomes were found to specifically target host “super enhancers” that have a strong affinity for the binding of transcriptional coactivators in order to facilitate greater EBV gene expression and cancer proliferation [21]. Similar to other herpesviruses, the EBV life cycle alternates between latent and lytic states [22]. In immunocompetent individuals, EBV is typically found in a latent, asymptomatic state. Disturbances of the host immune system can stimulate viral reactivation [23]. These includes stressors such as oxidative stress, co-infection with viruses such as CMV or HPV, and immunosuppressive or chemotherapeutic treatments and stem cell transplantation. A comprehensive review of factors that can stimulate EBV reactivation was discussed by Sausen et al. in a separate review [23].

EBV is associated with a host of diseases, including but not limited to Sjögren’s syndrome [24], systemic lupus erythematosus [24], rheumatoid arthritis [24], hairy leukoplakia [25], Alzheimer’s [26], Parkinson’s [26], and acute cerebellar ataxia [26]. Additionally, a recent study of greater than 10 million young adults demonstrated that EBV infection resulted in a 32-fold increased risk of developing multiple sclerosis (MS) [27]. In this study, neurofilament light chain, a marker of neuroaxonal degeneration, increased following EBV infection, indicating that EBV may be a driving factor in the pathogenesis of MS [27]. This is reminiscent of virus-induced animal models (e.g., Theiler’s murine encephalomyelitis virus model) of demyelinating diseases including MS. [28] 

**Figure 1:**
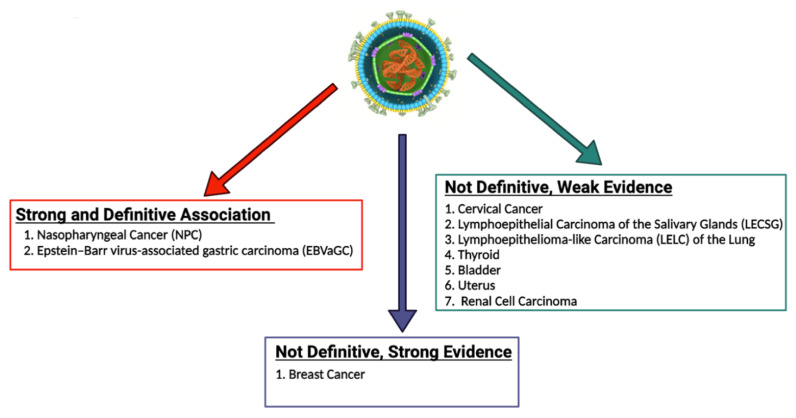
EBV-associated epithelial malignancies. EBV is associated with numerous malignancies with varying degrees of evidence. There is numerous evidence linking EBV to both nasopharyngeal carcinoma and gastric carcinoma. With regard to breast cancer, some studies provide strong evidence that EBV plays a role in the pathogenesis of breast cancer, while some studies call for more definitive evidence to be published. EBV has been less definitively associated with numerous other epithelial malignancies, including cervical cancer, lymphoepithelial carcinoma of the salivary glands, lymphoepithelioma-like carcinoma of the lung, thyroid cancer, bladder cancer, uterine cancer, and renal cell carcinoma [34,35,36,37,38,39,40,41,42,43,44,45,46]. The role of EBV in the pathogenesis of these cancers should be further explored and definitively established.

Since Epstein and Barr first discovered EBV in Burkitt lymphoma (BL) cells in 1964, a myriad of other malignancies have been both strongly and causally linked to EBV [29]. These malignancies can be categorized as those which are lymphoproliferative and those which are epithelial in nature [30]. In addition to BL, lymphoproliferative diseases associated with EBV include Hodgkin lymphoma (HL), diffuse large B cell lymphoma (DLBCL), and extranodal T/NK cell lymphoma, as well as the rarer plasmablastic lymphoma (PBL) and primary effusion lymphoma (PEL) [31]. Epithelial malignancies with a well-known association with EBV include gastric cancer (GC) and nasopharyngeal cancer (NPC) [32]. Additionally, a recent systematic review and meta-analysis found that there is a strong statistical relationship between EBV infection and the risk of developing breast cancer [33]. Other epithelial malignancies with a weaker correlation to EBV include lymphoepithelial carcinoma of the salivary glands (LECSG), lymphoepithelioma-like carcinoma of the lung (LELC), renal cell carcinoma, thyroid cancer, cervical cancer, bladder cancer and leiomyomas/leiomyosarcomas in immunocompromised patients [34,35,36,37,38,39,40,41,42,43,44,45,46]. Figure 1 below lists these epithelial malignancies associated with EBV. It is important to note that EBV infection does not lead to malignant transformation of normal epithelial cells, raising uncertainty about the causal role of EBV in the oncogenesis of these cancers [47].

This review begins with a brief discussion on the latency and reactivation of EBV. Then, we discuss the major epithelial malignancies associated with EBV as well as the latest methods of treatment for these conditions. We also mention several epithelial malignancies that are believed to be associated with EBV but need further studies to confirm the relationship.

## 2. EBV Latency

EBV latency, and more particularly the proteins expressed during this phase of the viral cycle, is heavily implicated in EBV-mediated oncogenesis [48,49]. The proteins ultimately expressed during latent infection varies based on the latency type. In type I latency, infected cells express EBNA1, EBV-encoded small RNA (EBER), and BamHI fragment A rightward transcripts (BART) transcripts. In type IIa latency, infected cells express everything seen in type I latency as well as latent membrane proteins (LMP) 1 and 2. Type IIb latency resembles type IIa latency, but features the expression of EBNA2, EBNA3, and EBNA-leader protein (LP) instead of LMP 1 and 2. Type III latency includes the expression of EBNA1, 2, 3A, 3B, and 3C, EBNA-LP, LMP 1 and 2, EBER 1 and 2, and the microRNAs (miRNA) miR-BHRF1 and miR-BART3. A type 0 latency has also been described in which only EBERs are expressed.

EBV-related cancers are associated with specific latency patterns [50]. For example, EBVaGC is associated with latency type I or II [51] while nasopharyngeal cancer is associated with type II latency [52]. Among the EBV-associated hematologic malignancies, Burkitt lymphoma is typically characterized by type I latency [50], diffuse large B cell lymphoma most frequently expresses a type II latency (although it is less often associated with type III latency) [50], and both classic [53] and nodular lymphocyte-predominant (NLPHL) [54] Hodgkin lymphoma typically express type II latency. Notably, there is some evidence to suggest that certain cancers can express non-canonical latency patterns ([55,56]).

More details about these patterns can be found elsewhere [57,58]. Table 1 summarizes EBV latency expression patterns and Table 2 reviews which patterns of latency are associated with which malignancy.

The ability of EBV to transform B cells has long been known [59,60], and latent gene products have been implicated in the transformation process [60,61,62]. B cell transformation results in significant differences in B cell gene organization and expression. For example, Hernando et al. found that B cells transformed by EBV displayed altered methylation markings and endonuclease activity when compared to non-transformed cells [63]. These changes were noted to affect thousands of genes and were not seen in B cells whose proliferation was induced with CD40/IL4 [63]. Of particular note, EBV infection leads to hypermethylation, and therefore decreased expression, of tumor suppressor genes when compared to naïve B cells [64]. An RNA sequencing analysis of gene expression in primary human resting B lymphocytes infected with the EBV strain B95.8 revealed changes in gene expression in nearly 3700 genes, including changes in 94% of the genes required for lymphoblastoid cell line (LCL) growth and survival [65].

### EBV Latent Proteins and Oncogenesis

Gene products expressed during EBV latency play key roles in oncogenesis. LMP1 is a highly oncogenic protein that mimics CD40 signaling, leading to stimulation of multiple pathways [66], including nuclear factor kappa beta (NF-κB) [66,67]. It exerts its oncogenic properties through multiple mechanisms in a variety of tumor types. For example, LMP1-mediated p53 degradation and subsequent enhanced tumorigenesis [68] has been noted in several cancer lines [69]. LMP1 also activates PI3K/AKT, and the combination of PI3K/AKT and NF-κB activation has been shown to inhibit apoptosis in lymphoma patients [70,71]. In nasopharyngeal cancer cells, LMP1 alters miRNA expression, which may promote tumor formation [48]. In addition to its role as a B cell receptor mimic, LMP2A impairs apoptosis and cell cycle checkpoints and works synergistically with oncogenes to enhance survival and proliferation [49].

**Figure 2:**
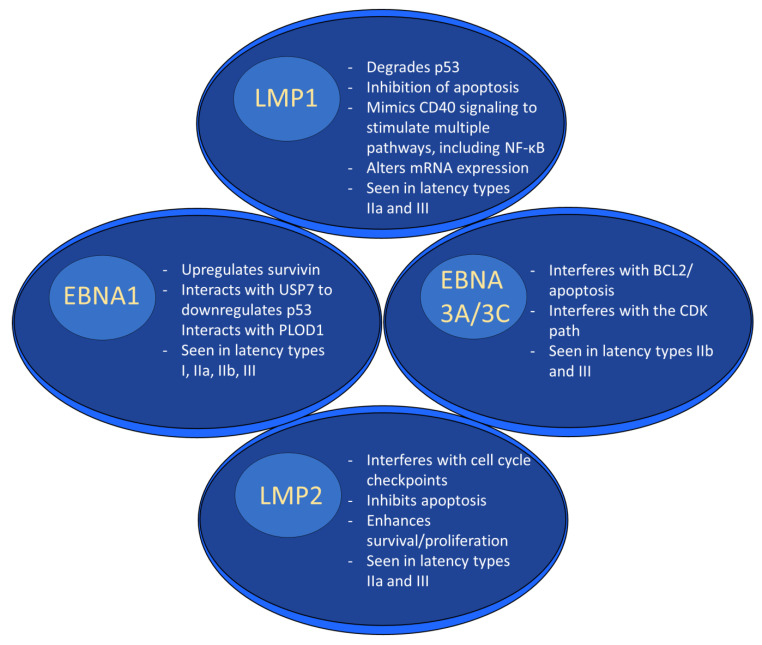
EBV latent proteins and oncogenesis. EBV latent proteins play numerous roles in facilitating oncogenesis. For example, LMP1 degrades p53, alters mRNA expression and stimulates multiple pathways through mimicking CD40 signaling. EBNA1 upregulates survivin, downregulates p53, and interacts with PLOD1. EBNA3A/3C interferes w/BCL2/apoptosis and interferes with the CDK pathway. LMP2 interferes with the cell cycle, inhibits apoptosis, and enhances survival/proliferation.

EBNA1 is another latent protein implicated in tumor formation. In B cell lymphoma, it has been shown to upregulate an anti-apoptotic protein named survivin [72,73]. In nasopharyngeal carcinoma, ENBA1 can inhibit NF-κB through inhibition of IKKα and β, which promotes development of squamous cell carcinoma by stimulating tissue hyperplasia [74]. EBNA1 interacts with ubiquitin-specific protease 7 (USP7) to decrease P53 levels [75]. In addition, it was recently shown that some EBNA1 variants, particularly those with the amino acid substitution T85A, can more easily bind the cellular protein Procollagen-lysine, 2-oxoglutarate 5-dioxygenase 1 (PLOD1) [76], which is associated with gastric cancer [77]. Moreover, PLOD1 overexpression carries a poorer prognosis in gastric cancer [78]. The EBNA3 family of latent genes, including EBNAs3A, 3B, and 3C, are intriguing in that they play opposing roles in oncogenesis, with EBNA3A and 3C promoting cancer formation and EBNA3B suppressing it [79]. EBNA3A and 3C interact to induce tumor formation through a variety of mechanisms, including interfering with the BCL2/apoptosis and cyclin dependent kinase (CDK) pathways [80]. As was mentioned above, EBNA3B acts as a tumor suppressor; indeed, murine infection by EBV strains lacking EBNA3B result in aggressive, immuno-evasive diffuse large B cell lymphoma (DLBCL) [81]. Infected cells secreted lower levels of the T cell chemoattractant CXCL10, which inhibited T cell recruitment and killing of infected cells. Notably, human B cell lymphomas were shown to have altered EBNA3B expression [81]. The role of selected EBV proteins has been summarized in Figure 2 below.

## 3. EBV and Epithelial Malignancies

In addition to infection of lymphoid cells, EBV can also infect epithelial cells as discussed throughout this review. While EBV readily infects B cells in vitro and transforms them into proliferative lymphoblastoid cell lines, EBV does not as readily infect human epithelial cells in vitro [82]. This discrepancy has made studying the interaction between EBV and epithelial cell lines more difficult and has hindered past research efforts [82]. However, recent successes, such as EBV being replicated in stratified tissues, have allowed better understanding of the mechanism by which EBV infects epithelial cells [83]. Indeed, this model was used by Yu et al. to study EBV epithelial cells from the nasopharynx to better understand how NPC develops [83]. This study demonstrated that EBV disrupted epithelial integrity and that noncancerous stratified pharyngeal epithelia were susceptible to EBV infection with their differentiation leading to lytic replication [83]. Although infection with EBV may not be the initiating factor for epithelial cell tumors, it has been shown to change host gene expression to better facilitate cancer growth by promoting cell survival, proliferation, invasion, epithelial–mesenchymal transition (EMT), angiogenesis and immune evasion [84]. In a recent study by Xiang et al., EBV infection was associated with the expression of endothelial phenotypes promote a vasculogenic-like network that could promote growth in EBV-infected epithelial malignancies [84]. Additionally, EBV infection was found to induce F3-mediated platelet aggregation, which inhibits the ability of NK cells to suppress tumors [85].

EBV entry into epithelial cells is governed by a slightly different mechanism than its entry into B cells. In B cells, a complex composed of gp42/gH/gL is required for entry. In epithelial cells, the gH/gL complex is thought to be essential, but gp42 is not necessary because epithelial cells lack HLA class II molecules [61,62]. Interestingly, it has been shown that the cell type in which EBV is grown impacts its future tropism [14]. Viruses grown in HLA class II positive cells, such as B lymphocytes, have a relatively greater proportion of EBV virions expressing the gH/gL complex lacking gp42 and have a subsequent increase in their affinity for epithelial cells. This occurs because gp42-containing trimers interact with HLA class II molecules that are en route to the cell membrane. Transportation occurs in vesicles containing cysteine proteases, which degrade the gp42/gH/gL complexes and shift the ratio to favor gH/gL complexes [62].

No specific treatments are associated with EBV-positive gastric cancers [86]; however, there have been recent advances in targeting LMP1 in EBV-positive NPC using DNAzymes that specifically target the LMP1 mRNA [87]. Additional methods of targeting EBV-positive NPC include methods such as targeting the DNA binding/dimerization site of EBNA1 and inhibition of LMP1 [88]. Understanding the interplay between EBV and epithelial cancers is of critical importance to further promote the development of treatments targeting malignancies that are positive for EBV.

## 4. EBV and Gastric Carcinoma

According to a recent meta-analysis, EBV is associated with 8.77% of gastric carcinomas, including 10.83% of cases in males and 5.72% of cases in females [89]. EBV-associated gastric cancer (EBVaGC) expresses type I and II latency patterns [63]. Genetic analysis showed that EBNA1 was the most commonly expressed protein, and LMP1 and LMP2A were also detected. Interestingly, 18 lytic genes were also expressed in these cancers, including both early and late EBV lytic proteins. This expression pattern is not consistent with either lytic or abortive lytic gene expression [64]. A meta-analysis demonstrated that EBNA1 was detected in greater than 98% of EBVaGC and that LMP2A was expressed in 53.8% of tumors. LMP1 and LMP2B were only noted in 10% of cases [65].

Despite its near ubiquitous expression in EBVaGC, little is known about EBNA1′s role in oncogenesis [34]. However, EBNA1 expression in EBV-negative gastric carcinoma resulted in increased tumorigenicity and growth rate as well as worsened histopathologic grading in xenograft-negative mice [90]. SCM1 gastric carcinoma cells expressing EBNA1 were also rendered less susceptible to cisplatin therapy [90]. These findings were not reproduced in TMC1 gastric carcinoma cells, which have a mutant form of P53 [90]. Cheng et al. found these findings to be consistent with a model in which EBNA1 competitively reduces p53 binding to USP7. This ultimately lowers p53 levels [75].

Lower p53 levels are associated with tumorigenesis [68]. EBNA1 has long been known to interact with USP7 [69]; indeed, EBNA1 occupies the same binding pocket as p53 and makes more extensive contact with USP7 [21]. In addition to confirming that EBNA1 reduces p53 activation and apoptosis, Sivachandran et al. demonstrated that EBNA1 interferes with promyelocytic leukemia nuclear bodies, which are important in apoptosis, p53 activation, and tumor suppression in AGS GC cells [70].

More is known about LMP2A’s role in gastric carcinogenesis. The Ras association domain family (RASSF) is a group of proteins that function as tumor suppressors [91]. Notably, it is a target of epigenetic silencing in multiple tumor lines [48], including gastric cancer [49]. RASSF10 promoter hypermethylation and subsequent downregulation of RASSF10 expression was noted in 6 of 8 GC cell lines examined [49]. Mechanistically, it was shown that LMP1 facilitates hypermethylation of the RASSF10 gene by recruiting DNA methyltransferase (DNMT1) [3]. Both sets of experiments demonstrated that overexpression of RASSF10 inhibited tumor formation [3,49].

Vasculogenic mimicry is a mechanism by which tumor cells secure a blood supply in the absence of angiogenesis or endothelial cells (reviewed in [72]). Recently, it was discovered that EBV promotes vasculogenic mimicry in epithelial cancers, including NPC and GC [84]. Specifically, LMP2A overexpression resulted in increased activation of the AKT/HIF-1α signaling pathway in NPC cell lines [84]. When examined in the context of GC, the EBV+ GC cell lines GT38 and GT39 formed tubes on 3D Matrigel culture, unlike EBV− AGS cells. Like the NPC cells, GT38 and GT39 demonstrated increased flux through the nuclear HIF-1α pathway, indicating EBV promotes vasculogenic mimicry in GC [74]. Another mechanism through which EBV promotes vasculogenic mimicry is through upregulation of the chemokine CXCL8. Addition of recombinant CXCL8 to GC cells led to vasculogenic mimicry and increased cell migration, while knockdown reduced tube formation on Matrigel culture. This effect is mediated via NF-κB signaling [75].

EBV is known to encode a wide array of miRNAs. While many of their actions are poorly understood, studies have examined their contributions to oncogenesis [76]. Microarray analysis demonstrated that 40 miRNAs were expressed in EBVaGC, all coming from miR-BART cluster 1 or cluster 2 [92]. Yoon et al. further examined the role of one such miRNA, miR-BART-5p, in GC [92]. This miRNA was inversely associated with levels of the PIAS3 protein in multiple GC cell lines. Reduced PIAS3 augments signaling through signal transducer and activator of transcription 3 (STAT3), which ultimately leads to the upregulation of programmed death-ligand 1 (PD-L1) [92]. Removing PD-L1 expression in SNU601 cells transfected with miR-BART5-5p via small interfering RNA (siRNA) resulted in significantly increased apoptotic levels. In addition, abrogation of PD-L1 upregulation resulted in decreased cell proliferation, invasion, and migration [77]. Decreased levels of AT-rich interaction domain 1A (ARID1A) are associated with the initiation of oncogenesis in EBVaGC, although this loss precedes EBV infection [78]. MiR-BART11-3p and miR-BART-12 were found to decrease ARID1A levels as shown by Western blotting [93]. ARID1A mRNA was not changed, consistent with posttranscriptional regulation. Notably, the ARID1A promotor was not hypermethylated [93].

While it is clear that EBV-encoded miRNAs play a role in EBVaGC oncogenesis, they have anti-oncogenic properties as well [94]. For example, EBV-encoded miRNAs manipulate the cell cycle by interfering with eukaryotic translation initiation factor 4E (eIF4E) signaling, which decreases cell proliferation [94]. When miR-BART11-p3 mimics were utilized, they increased the luciferase activity of wild-type but not mutant eIF4E 3′-UTR, and transfection of the miR-BART11-p3 mimic resulted in decreased eIF4E 3 expression [94]. miR-BART11-p3 mimic transfection reduced cell proliferation [94]. Wang et al. suggested that this inhibition of eIF4E 3 may partially explain the better prognosis given to EBVaGC [94].

Interestingly, when EBV-negative AGS GC cells were transfected with LMP2A, expression of HLA class I was inhibited [95]. This effect was eliminated when a mutated variant of LMP2A was expressed in place of wild-type LMP2A. Further analysis demonstrated that this effect was mediated through the sonic hedgehog pathway, and specifically through Gli1 [95]. Deb Pal et al. postulated that this may be one mechanism that allows EBVaGC to evade the immune system, thus promoting tumor survival [95]. PD-L1 expression is another mechanism through which EBVaGC evades the immune system. Indeed, analysis of the gastric cancer cell lines NCC24, SNU719, AGS, NUGC3, and YCCEL1 revealed elevated PD-L1 expression [96]. EBVaGC cells expressing PD-L1 suppressed T cell proliferation [96].

### Gastric Cancer Therapies

Treatment for EBVaGC can include chemotherapy, radiation, and surgical resection, with the latter being necessary to effectuate a cure [97]. In patients for whom systemic therapy is warranted, the current standard chemotherapy is the FLOT (fluorouracil, leucovorin, oxaliplatin, and docetaxel) regimen [98].

EBVaGC overexpresses PD-L1 [99]. A recent clinical trial assessed the efficacy of the humanized monoclonal anti-PD-1 antibody pembrolizumab compared to pembrolizumab with chemotherapy, and to chemotherapy alone. Pembrolizumab was found to be noninferior to chemotherapy in terms of overall survival, as patients treated with pembrolizumab had an average survival of 10.6 months vs. chemotherapy, which had an average survival of 11.1 months [100]. In patients with high PD-L1 expression, defined as a PD-L1 combined positive score of >10, survival with pembrolizumab increased to 17.4 months [100]. Chemotherapy survival in this cohort was 10.8 months, although this difference was not significant enough to demonstrate superiority to chemotherapy [100]. Patients treated with pembrolizumab plus chemotherapy had a median survival of 12.5 months [100]. Patients in this group with high PD-L1 expression had a median survival of 12.8 months [100]. Notably, patients treated with pembrolizumab alone (54.3%) experienced fewer adverse events than patients receiving chemotherapy (91.8%) or both pembrolizumab and chemotherapy (94.0%) [101]. Nivolumab is another PD-1 inhibitor that has been examined in gastric cancer. A comparison between nivolumab and nivolumab + chemotherapy showed improvements in both overall survival and progression free survival in patients treated with nivolumab and chemotherapy when compared to chemotherapy alone [102]. There was a higher rate of severe adverse events in the dual therapy group, with 59% of patients treated with nivolumab + chemotherapy experiencing a grade 3-to-4 adverse event when compared to only 44% of patients treated with chemotherapy alone [102]. Interestingly, patients who were treated with anti-PD-1 therapy responded better to chemotherapy with the antimicrotubular class of drug known as taxanes, taxanes with ramucirumab, or the alkaloid known as irinotecan than patients who did not have prior treatment targeting PD-1 [103]. Overall response rates to chemotherapy were 44.6% in those previously treated with anti-PD-1 treatment and 19.6% in anti-PD-1 naïve patients [103].

ARID1A expression is frequently lost in EBVaGC. While this loss of expression precedes EBV infection [104], it does facilitate EBVaGC carcinogenesis [78]. Research has examined the potential of targeting ARID1A mutations in gastric cancer. Treating gastric organoids with the epigenetic inhibitor TP064 and the p53 agonist Nutlin-3 decreased organoid viability significantly more than organoids with retained ARID1A expression, potentially through synergistic activation of p53 signaling [105]. Enhancer of zeste homolog 2 (EZH2) is a subunit of polycomb repressive complex 2 (PRC2) that regulates gene expression, at least in part by trimethylation of Lys-27 in histone 3 (H3K27me3). It has recently gained attention as a treatment target in cancer therapy [106]. Treating ARID1A-WT MKN7 and ARID1A-MUT NUGC-3 cells with EZH2 inhibitors resulted in a more significant decrease in viability in the ARID1A mutated cells, potentially due to a decrease in PI3K/AKT signaling [107].

## 5. EBV and Nasopharyngeal Cancer

NPC is a type of malignancy that originates from the epithelium of the nasopharynx, with 5-year survival rates of 83.7% for localized NPC, 75% for regional NPC and 62.2% for distant NPC. [108]. NPC is an uncommon epithelial tumor that has a unique ethnic and geographical distribution. The World Health Organization (WHO) estimated that there were approximately 129,000 new NPC cases reported worldwide in 2018 [109]. NPC is endemic in indigenous populations in East and Southeast Asia, the Arctic, and North Africa and the Middle East [110]. In Asia, NPC is most commonly observed in people of Chinese descent, specifically the Cantonese [111]. In the Arctic, persons of Innuit origin experience higher rates of NPC, while those of Arab descent experience higher rates in North Africa [112,113]. Interestingly, studies following trends of NPC development in immigrants found that overall rates of NPC were higher in people emigrating from areas of high risk compared to native populations. This elevated risk compared to the native population was still present in second- and third-generation immigrants [114]. In addition to ethnic and geographical factors, EBV infection is heavily associated with NPC development, with over 95% of tumors being EBV-positive in areas of high incidence [115]. Other factors that play a role in development of NPC include dietary habits, lifestyle, host immunity and environmental factors [116].

The association between EBV infection and NPC was first established through the detection of high concentrations of serum antibodies against EBV antigens, including viral capsid antigen (VCA) and early antigen diffuse component (EAd/BMRF1) [117]. Later, elevated levels of circulating cell-free EBV DNA were detected in patients with NPC and subsequentially shown to have a strong correlation with NPC tumor stage and overall survival rates. To date, circulating EBV DNA has been shown to be of clinical value in screening, prognostication, and surveillance for recurrent NPC [118]. In NPC, EBV exists in a latent state that is exclusively found in the tumor cells but absent from the surrounding lymphoid infiltrate. However, it should be noted that the malignant NPC cells are highly dependent on the adjacent carcinoma cells and prominent lymphoid stroma found in undifferentiated NPC [119].

LMP1 is expressed in NPC and is known to trigger several important signal transduction pathways such as NF-κB, ERK-MAPK, PI3K/AKT, JNK, JAK/STAT, and others involved in the growth and metastasis of tumor cells [66,120]. LMP1 has been shown to demonstrate oncogenic properties both in vivo and in vitro [121]. It regulates the expression of several downstream targets associated with cell growth, survival, epithelial-mesenchymal transition (EMT), migration, invasion and aerobic glycolysis, and immune evasion [122]. LMP1 suppresses NK and T cells and can be considered a critical therapeutic target due to its multiple oncogenic properties [122]. LMP1 is variably expressed in NPC tissues, with immunohistochemical (IHC) methods estimating expression rates to be between 20–60%. However, LMP1 is heavily implicated in contributing to the early stages of NPC pathogenesis as it is found in most premalignant or preinvasive NPC tissue samples [122]. NPC tumors expressing LMP1 were found to be more aggressive and invade lymph nodes more easily than those which were LMP1-negative [123]. Moreover, LMP1 was shown to contribute to radioresistance in NPC by promoting cell protective autophagy, thus leading to poorer rates of survival [124].

The LMP2 proteins (LMP2A and LMP2B) are another set of hydrophobic integral membrane proteins that are consistently detected in NPC [125]. While the role of LMP2B is less defined, LMP2A has been shown to play a key role in maintaining EBV latency [126]. LMP2A has been demonstrated to promote various oncogenic phenotypes by regulating multiple signaling pathways, such as PI3K/AKT, ERK and RhoA [88]. Additionally, LMP2A is known to support the function of LMP1 and contribute to malignant transformation of host cells by intervening with signaling pathways at multiple points and promoting proliferation, anti-apoptosis, and angiogenesis, as well as cell invasion and metastasis [127,128]. LMP2A also blocks BCR-mediated intracellular release of calcium and PTK cascades, resulting in inhibition of B-cell differentiation [129].

BamHI-A rightward frame 1 (BARF1) is a protein encoded by Bam H1-A that is secreted during latency in EBV-associated NPC [130]. BARF1 shares homology with the human colony stimulating factor 1 (hCSF-1) receptor (*c-fms* oncogene) and competes for its ligand, macrophage colony-stimulating factor (M-CSF) [131]. It plays a role in modulating immune cell growth and function by acting as an allosteric decoy receptor for hCSF1 and interfering with macrophage activation and the immune response [132,133]. Moreover, BARF1 was shown to play a role in suppression of apoptosis via activation of BCL-2 and upregulation of NF-κB, RelA, and cyclin D1 expression in NPC cells [127]. BARF1-negative EBV cells that were engineered to express BARF1 showed increased growth and tumorgenicity, thus supporting the role of BARF1 in NPC pathogenesis [132].

EBER1 and EBER2 are a set of two small non-polyadenylated, double-stranded RNAs that are the most abundant viral transcripts in latent EBV-infected cells [134]. EBERs contribute to EBV associated oncogenesis by modulating innate immunity in patients with NPC [135]. EBERs play a role in the regulation of LMP1 and LMP2, and therefore play a role in promoting latent EBV infection in cells [136]. EBERs may also interact with TLR3 and induce tumor cells to produce cytokines and recruit macrophages to promote the growth of NPC cells [137].

Alternatively spliced BARTs code for several oncogenic long noncoding RNAs (lncRNAs) and are highly expressed in NPC. BART lncRNAs localize within the nucleus of EBV-infected cells; however, their role in EBV latency and NPC has yet to be clarified [138]. Interestingly, EBV-encoded microRNAs, miR-BARTs, play a role in promoting cellular growth and proliferation, inhibiting cell apoptosis, and maintaining virus latency, as well promoting tumor metastasis and immune evasion [139]. Numerous miR-BARTs have been identified, and they compose 26–38% of the total cellular microRNAs in NPC tumors [140].

### Nasopharyngeal Cancer Therapies

NPC can have a 91–94% survival rate when diagnosed in its earliest stages (stages I–IIB) [140]. However, early-stage NPC is often asymptomatic; therefore, biomarkers such as circulating cell-free EBV DNA are used to detect NPC in high risk populations [141]. In a study performed by Chan et al., post-radiotherapy plasma EBV levels were found to be significantly correlated with hazards such as distant metastasis and death [142]. Chan et al. suggested that post-radiotherapy plasma EBV DNA be measured to determine risk clarification and treatment selection for adjuvant therapy in NPC [142]. Another method of detecting NPC involves the detection of EBV DNA in nasopharyngeal brush cytology. This method was found to have a sensitivity of 98.9%, a specificity of 99.3%, a positive predictive value (PPV) of 96.9% and a negative predictive value (NPV) of 99.7% for detecting NPC. Overall, this yielded better results than endoscopy [143]. Individuals with elevated plasma biomarkers are then evaluated via nasopharyngeal endoscopic exam. An abnormal finding or suspicion for NPC based on the endoscopic exam is followed by endoscopic-guided biopsy to confirm NPC [144].

Previously, platinum-based concurrent chemoradiotherapy was considered to be the standard of care for locoregionally advanced NPC [145]. The addition of the nucleoside analog known as gemcitabine in combination with the platinum analog known as cisplatin forms what is known as GP induction chemotherapy. In a study conducted by Zhang et al., GP induction therapy added to a standard chemoradiotherapy regimen was found to significantly improve recurrence-free survival as well as overall survival when compared to chemoradiotherapy alone in patients with locoregionally advanced NPC [145]. Patients in the Zhang et al. study who received the induction chemotherapy benefited from a higher rate of 3-year recurrence-free survival (85.3% compared to 76.5% for the standard-therapy group) [145]. In recent years, GP therapy has become the preferred first line treatment for patients with recurrent or metastatic NPC [146]. Recent studies have yielded promising results when comparing toripalimab (an antibody against PD-1) versus placebo in addition to gemcitabine and cisplatin therapy [147]. The addition of toripalimab to standard gemcitabine and cisplatin therapy increased progression-free survival as well as overall survival when compared to the addition of placebo to gemcitabine and cisplatin therapy [148]. In a study performed by Zhou et al., the cellular protein coding gene AKR1C1 was identified as a good prognostic factor for advanced-stage NPC [149]. Zhou et al. discovered that cisplatin sensitivity was increased in ARK1C1-silenced NPC cells and advocated for further studies to validate AKR1C1 as a marker for predicting response to cisplatin in patients with NPC [149]. In another study by Gao et al., the role of Brain-expressed X-linked 3 (BEX3), a CD271-receptor-associated protein, in NPC was investigated due to its upregulation in this disease [150]. Gao et al. also discovered that BEX3 was upregulated in response to cisplatin treatment and was also implicated in mediating cisplatin resistance in NPC cells [150].

Radiotherapy (RT) is considered the mainstay of treatment for non-disseminated NPC [151]. Intensity-modulated RT (IMRT) has been shown to provide more accurate dose delivery to targets and less toxicity to normal organs when compared to traditional RT [152]. In addition, a significant reduction in late effects such as xerostomia, trismus, and injury to the temporal lobe was reported in patients who underwent IMRT [153]. A recent meta-analysis performed by Luo et al. found IMRT to have increased overall survival, locoregional control rate, disease-free survival, and metastasis-free survival in comparison with traditional RT [154]. Particle therapy, including the use of protons and carbon ions, has also been recently used in the treatment of NPC. Proton therapy specifically is a promising approach for targeting locally advanced NPC as it avoids damage to neurological structures [154]. A systematic review by Lee et al. found that outcomes for patients undergoing proton therapy were similar to those who underwent IMRT, with 2-year local and regional progression-free survival rates between 84–100%, 2-year progression free survival between 75–88.9% and 2-year overall survival between 88–95% [155]. This review also noted that patients undergoing proton therapy required feeding tubes less frequently when compared to IMRT (20% versus 65%) and experienced lower rates of mucositis (46% versus 70%) [155].

Surgical options such as nasopharyngectomy represent another method of treating locally recurrent NPC. Recently, minimally invasive techniques such as endoscopic surgery have been utilized to help treat locally recurrent NPC. These results have yielded positive 5-year overall survival rates for recurrent type 1 (rT1) and recurrent type 2 (rT2) cases of recurring NPC [156]. A recent clinical trial performed by Liu et al. found that patients with locally recurring NPC had significantly improved survival rates when treated with endoscopic surgery as opposed to IMRT [157]. These results were also confirmed by Peng et al., who specifically compared IMRT with recurrent later-stage rT3 and rT4 NPC [158]. This meta-analysis added that endoscopic surgery was associated with higher rates of survival and lower overall complications when compared with IMRT [158].

## 6. EBV and Other Malignancies

In addition to EBVaGC and NPC, other epithelial cancers have been associated with EBV; however, their definitive association has not been confirmed. These malignancies include breast cancer, lymphoepithelial carcinoma of the salivary glands (LECSG), lymphoepithelioma-like carcinoma of the lung (LELC), renal cell carcinoma, thyroid cancer, cervical cancer, bladder cancer and leiomyomas/leiomyosarcomas in immunocompromised patients [34,35,36,37,38,39,40,41,42,43,44,45,46].

Worldwide, female breast cancer is the most commonly diagnosed cancer, with an estimated 2.3 million new cases in 2020 [159]. In the United States, breast cancer is the most commonly diagnosed cancer among women in the after skin cancer [160]. It is also the second leading cause of cancer deaths among women in the United States after lung cancer [160]. The link between EBV and breast cancer was first discussed by Labrecque et al. in 1995 [161]. Since then, the association has yet to be definitively concluded, with different studies suggesting different levels of association. 

One meta-analysis of 4607 cases from 27 countries indicated a high pooled prevalence of 26.37% among breast cancer patients [33]. Additionally, this meta-analysis attributed a 4.74-fold increase in breast cancer due to EBV infection [33]. Another meta-analysis conducted by Jin Q et al. analyzed sixteen studies comprising 1279 cases and 814 controls between 1995 and 2018. Their results determined that EBV infection had a significant association with increased breast cancer risk with an odds ratio of 4.75 (*p* = 0.01) [162]. Overall, EBV infection is more prevalent in breast cancer tissue samples when compared to controls.

Another recent study by Zhang et al. discussed a higher incidence of high-grade invasive breast cancer in patients with EBV infection when compared to those who were not previously infected [163]. This study also concluded that patients with EBV infection were more likely to experience negative prognostic factors such as higher rates of lymph node metastasis (59.7%), lymphovascular invasion (72.1%) and presentation with a worse clinical stage of either stage III or IV breast cancer (90.5%) [163].

A paper by Hu et al. suggested that EBV may play a role in the early formation of breast cancer via epithelial mesenchymal transition [164]. Their data suggested that EBV LMP1 triggers the activation of NF-κB through the activation of c-MET. Moreover, the authors suggested that EBV promotes oncogenesis via alteration of APOBEC3, which is an antiviral enzyme [164]. Hu et al. also concluded that EBV infection was correlated with adverse clinicopathological features [164]. Another proposed mechanism by which EBV infection promotes breast cancer is via activation of the HER2/HER3 oncogenes [165].

While some studies highlight the connection between EBV and breast cancer, other studies have failed to find traces of EBV in breast cancer tissue. In a study performed by Dowran et al., 300 breast biopsy tissues from both malignant and benign tumors did not show any positivity for EBV DNA fragments [166], while another study performed by Salih et al. suggested that EBV does not play a vital role in the pathogenesis of breast cancer but was associated with lymph node metastasis, age group and estrogen receptor presence. The study also determines that a definitive link between EBV and breast cancer can not be established [167].

The relationship between EBV and other rarer cancers is even more difficult to define due to the limited number of published studies. LECSG is one such cancer. A study by Whaley et al. examined the relationships between LECSG and EBV and found that while LECSG may express EBER, LECSG was not associated with non-endemic patients [168]. With regard to renal cell carcinoma, few studies have been conducted to examine the role of EBV in renal cell carcinoma pathogenesis. Shimakage et al. described the presence of EBV in various types of renal cell carcinoma tissues [38]. However, an earlier study performed by Kim et al. only found signs of EBV infection in 5/73 (6.8%) of RCC tissue samples [39]. More recently, a study conducted by Farhadi et al. concluded that EBV did in fact play a role in the development of renal cell carcinoma through the activation of the NF-κB p65 signaling pathway [37]. The authors of these studies are in agreement that larger-scale studies are needed to more clearly define the relationship between EBV infection and renal cell carcinoma.

The relationship between thyroid cancer and EBV was described in a review conducted by Almeida et al. [169]. The authors discussed that the incidence of EBV in thyroid specimens can widely vary depending on the method of detection used to examine tissues and the populations the tissues are collected from. However, the authors concluded that there was a relationship between EBV and thyroid cancer and that further studies are needed to confirm this relationship [169]. With regard to cervical cancer, current evidence proposes that a causal relationship may exist between EBV and cervical cancer pathogenesis [43]. This is reiterated by a meta-analysis performed by de Lima et al. which pointed to the a positive association between pooled EBV prevalence and lesion grade in cervical epithelia, a high prevalence of EBV in malignant lesions, an increased odds ratio associated with EBV as a risk factor (OR = 4.01 [1.87–8.58]; *p* < 0.001), and the existence of EBV(+) HPV(−) carcinomas as reasons why EBV should be considered a risk factor for cervical cancer [42]. The authors of this meta-analysis also discuss their findings that latent oncoproteins such as EBNA1, EBNA2, LMP1 and EBERs were expressed in cervical tumors and that there was an association of EBV with the integration of high-risk HPV DNA in malignant tissue samples [42].

Very few studies have examined the relationship between EBV and bladder cancer. One such study is an investigation conducted by Abe et al. which detected EBER-expressing lymphocytes in 26 out of 39 bladder cancer cases (66.7%) [45]. However, the limited amount of data available led the authors to question the role EBV has in bladder cancer pathogenesis [45]. The first study linking leiomyomas/leiomyosarcomas in immunocompromised patients to EBV was conducted by KL McClain et al. in 1995 [46]. Since then, few articles have been written discussing the relationship. One such paper written by Magg et al. discussed that smooth muscle tumors should be screened for the presence of EBV and that patients presenting with EBV-positive smooth muscle tumors should be screened for possible primary immunodeficiency disorders [170].

There is also an interesting association between EBV and hepatocellular carcinoma, although supportive evidence is not conclusive [171]. Indeed, a meta-analysis of 918 patients showed that the prevalence of EBV was 9.35 times higher in hepatobiliary cancer than in healthy liver and duct tissue. The authors concluded that EBV may be a risk factor in the pathogenesis of hepatobiliary cancer [171]. Interestingly, EBV positivity was much more strongly associated with hepatocellular carcinoma with immune cell stroma than conventional hepatocellular carcinoma (74.5% vs. 4.6%, respectively) [172]. The corresponding number of tumor-infiltrating lymphocytes (TILs) per high-powered field were 10.5 ± 26.4, and 0.2 ± 1.0, respectively. Tumor PD-L1 expression was associated with the number of EBV-positive TILs [172]. EBV positivity was associated with better recurrence-free survival, although this trend was reversed in hepatocellular carcinoma with higher numbers of EBV+ TILs. In addition, T cell exhaustion was more prevalent in samples with high density EBV+ TILs [172]. The treatments for these cancers have been summarized in Table 3.

## 7. Conclusions

EBV is an oncogenic virus that is ubiquitous in our society. Indeed, EBV and its associated malignancies are responsible for a significant cancer burden worldwide [192]. It is clear that more research is needed to develop better treatment approaches. Indeed, exciting avenues of research are already being explored in the form of EBV-specific vaccines [193]. In addition, new research is examining the therapeutic potential of immune therapy targeting specific EBV antigens [194,195].

In this review, we discussed the latent stage of EBV and how it plays a role in the progression of epithelial associated malignancies. Furthermore, we discussed the two main epithelial cancers that have been strongly linked to EBV, EBVaGC and NPC, in addition to the other weakly associated malignancies and the most recent treatments available. While other malignancies may have a more causal link to EBV, these relationships must be further explored to better understand their pathogenesis.

## Figures and Tables

**Table 1:** Patterns of EBV protein expression during latency.

	Latency 0	Latency I	Latency IIa	Latency IIb	Latency III
EBNA1	−	+	+	+	+
EBNA2	−	−	−	+	+
EBNA3	−	−	−	+	+
EBNA-LP	−	−	−	+	+
LMP1	−	−	+	−	+
LMP2	−	−	+	−	+
BARTs	−	+	+	+	+
EBERs	+	+	+	+	+

Table 1 summarizes the EBV protein expression seen in each type of latency. “+” indicates the protein is expressed, while “−” indicates that the protein is not expressed.

**Table 2:** EBV latency patterns and associated malignancies.

Latency Type	Associated Malignancies
Latency I	EBVaGC, Burkitt lymphoma
Latency II	EBVaGC, NPC, DLCBL, classic Hodgkin’s lymphoma, NLPHL
Latency III	DLBCL

Table 2 provides an overview of which malignancies are associated with which patterns of EBV latent gene expression.

**Table 3:** Cancers associated with EBV and their treatments.

Cancer Type	Treatment Options	References
EBV-associated gastric cancer	Chemotherapy, radiation, and surgical resection, fluorouracil, leucovorin, oxaliplatin, and docetaxel or fluoropyrimidine and oxaliplatin, pembrolizumab, enhancer of zeste homolog 2 (EZH2) inhibitors	[97,100,107]
Nasopharyngeal carcinoma	Surgery, platinum-based concurrent chemoradiotherapy, gemcitabine and cisplatin induction chemotherapy + toripalimab, intensity-modulated (IMRT), particle therapy	[145,148, 152,154]
Breast cancer	Neoadjuvant chemotherapy, doxorubin and cyclophosphamide followed by paclitaxel (AC-T), docetaxel, paclitaxel, selective estrogen receptor modulators (SERMs), (tamoxifen, raloxifene, toremifene), aromatase inhibitors (non-steroidal anastrozole and letrozole and steroidal exemestane), (SERD), selective estrogen receptor degrader (fulvestrant), cyclin D kinase 4/6 inhibitors (palbociclib, ribociclib and abemaciclib), PI3K/Akt/mTOR pathway inhibitors, mTOR inhibitor (everolimus), PI3K inhibitor (alpelisib), lumpectomy, whole breast irradiation, mastectomy	[173,174,175]
Lymphoepithelial carcinoma of the salivary glands	Surgery (parotidectomy or submandibular gland excision) and induction chemotherapy, concurrent therapy, postoperative radiotherapy	[176,177,178]
Lymphoepithelioma-like carcinoma of the lung	Surgery, cisplatin-based chemotherapy, pembrolizumab nivolumab, arezolizumab, nivolumab and gemcitabine, other combination therapies	[179,180,181]
Renal cell	Pembrolizumab and axitinib, cabozantinib, pazopanib, bevacizumab, temsirolimus, surgery, cytoreductive nephrectomy	[182,183]
Thyroid	Surgical resection, TSH suppression, radioiodine therapy, pembrolizumab, vemurafenib, dabrafenib, selumetinib, everolimus, sorafenib, lenvatinib, pazopanib and treametinib	[184]
Cervical	Conization, hysterectomy, radiotherapy	[185]
Bladder	Trans-urethral resection of bladder tumor (TURBT), chemotherapy, induction intravesical therapy, induction BCG	[186]
Leiomyomas/leiomyosarcomas	EBV-CTL therapy, antiretroviral therapy, reduction of immunosuppressive treatment, surgery	[170]
Hepatocellular carcinoma	Surgical resection, radiation therapy, chemo/immunotherapy (doxorubicin, FOLFOX [folinic acid, fluorouracil, and oxaliplatin], sorafenib, regorafenib, atezolizumab + bevacizumab, others), image-guided tumor ablation, transcatheter arterial chemo/radioembolisation, combination therapies (ie systemic + intra-arterial treatment),	[187,188,189,190,191]

## Data Availability

All of the studies reported on in this review were collected from PubMed and Google Scholar.
